# Defining the Transcriptional Landscape during Cytomegalovirus Latency with Single-Cell RNA Sequencing

**DOI:** 10.1128/mBio.00013-18

**Published:** 2018-03-13

**Authors:** Miri Shnayder, Aharon Nachshon, Benjamin Krishna, Emma Poole, Alina Boshkov, Amit Binyamin, Itay Maza, John Sinclair, Michal Schwartz, Noam Stern-Ginossar

**Affiliations:** a Department of Molecular Genetics, Weizmann Institute of Science, Rehovot, Israel; b Department of Gastroenterology, Rambam Health Care Campus and Bruce Rappaport School of Medicine, Technion, Institute of Technology, Haifa, Israel; c Department of Medicine, Addenbrooke’s Hospital, University of Cambridge, Cambridge, United Kingdom; University of California, Irvine

**Keywords:** cytomegalovirus, gene expression, latency, single-cell RNA-seq, transcriptome

## Abstract

Primary infection with human cytomegalovirus (HCMV) results in a lifelong infection due to its ability to establish latent infection, with one characterized viral reservoir being hematopoietic cells. Although reactivation from latency causes serious disease in immunocompromised individuals, our molecular understanding of latency is limited. Here, we delineate viral gene expression during natural HCMV persistent infection by analyzing the massive transcriptome RNA sequencing (RNA-seq) atlas generated by the Genotype-Tissue Expression (GTEx) project. This systematic analysis reveals that HCMV persistence *in vivo* is prevalent in diverse tissues. Notably, we find only viral transcripts that resemble gene expression during various stages of lytic infection with no evidence of any highly restricted latency-associated viral gene expression program. To further define the transcriptional landscape during HCMV latent infection, we also used single-cell RNA-seq and a tractable experimental latency model. In contrast to some current views on latency, we also find no evidence for any highly restricted latency-associated viral gene expression program. Instead, we reveal that latency-associated gene expression largely mirrors a late lytic viral program, albeit at much lower levels of expression. Overall, our work has the potential to revolutionize our understanding of HCMV persistence and suggests that latency is governed mainly by quantitative changes, with a limited number of qualitative changes, in viral gene expression.

## IMPORTANCE

Human cytomegalovirus is a prevalent pathogen, infecting most of the population worldwide and establishing lifelong latency in its hosts. Although reactivation from latency causes significant morbidity and mortality in immunocompromised hosts, our molecular understanding of the latent state remains limited. Here, we examine the viral gene expression during natural and experimental latent HCMV infection on a transcriptome-wide level. In contrast to the classical views on herpesvirus latency, we find no evidence for a restricted latency-associated viral gene expression program. Instead, we reveal that latency gene expression largely resembles a late lytic viral profile, albeit at much lower levels of expression. Taken together, our data transform the current view of HCMV persistence and suggest that latency is mainly governed by quantitative rather than qualitative changes in viral gene expression.

## INTRODUCTION

Human cytomegalovirus (HCMV) is a ubiquitous pathogen that, like all herpesviruses, can establish latent infection that persists for the lifetime of the host. In healthy individuals, infection rarely causes any significant clinical symptoms, due to a robust immune response (1, 2). In contrast, primary infection or reactivation from latency can result in serious and often life-threatening disease in immunocompromised individuals (3–5). Latent infection is, therefore, a key part of viral persistence, and latently infected cells are a clear threat when the immune system is suppressed. Despite this, our molecular understanding of the HCMV latency state is still limited.

HCMV is tightly restricted to humans; however, in its host it has extremely wide cell tropism (6), and many kinds of cells can be productively infected, including fibroblasts, epithelial cells, and smooth muscle cells (7). In contrast, latent infection was so far characterized only in cells of the early myeloid lineage, including CD34^+^ hematopoietic progenitor cells (HPCs) and CD14^+^ monocytes (8). It was further established that terminal differentiation of HPCs and CD14^+^ monocytes to dendritic cells (DCs) or macrophages triggers virus reactivation from latency (9–13). This differentiation-dependent reactivation of latent virus is thought to be mediated by changes in posttranslational modification of histones around the viral major immediate early promoter (MIEP) (11, 14–17). These modifications drive the viral major immediate early (IE) gene expression, resulting in reactivation of the full viral lytic gene program cascade and the production of infectious virions (11). Thus, the cellular environment is a key factor in determining the outcome of HCMV infection.

During productive lytic infection, HCMV expresses hundreds of different transcripts and viral gene expression is divided into three waves of expression, IE, early, and late (6, 18, 19). The maintenance of viral genome in latently infected cells is thought to be associated with expression of a much smaller number of viral genes relative to lytic infection (20–25) in the general absence of IE gene expression. Due to their therapeutic potential, significant attention has been drawn to a few latency-associated viral gene products, but the possibility that additional viral transcripts contribute to latency regulation remains unclear.

The earliest studies that looked for latency-associated gene expression identified a number of transcripts arising from the MIEP region of HCMV, but no function was assigned to them (26–28). More systematic mapping of latency-associated transcripts was conducted with the emergence of microarray technology. Two studies detected a number of viral transcripts in experimentally latently infected myeloid progenitor cells (29, 30). The latent transcripts reported by these studies were not entirely overlapping, and yet these findings were used as a guideline for targeted efforts to identify latent gene products. Interrogating the viral transcriptome in natural persistent infection is highly challenging since viral genomes are maintained in extremely few cells, at very low copy numbers, and viral genes are expected to be expressed at low levels. Nevertheless, subsequent work detected a number of these transcripts during natural latency (22, 25), mainly using high-sensitivity approaches such as nested PCR, building a short list of viral genes that is generally accepted to represent a distinct transcriptional profile during latent infection. These genes include UL138, UL81-82ast (LUNA), and US28, as well as a splice variant of UL111A, which encodes a viral interleukin-10 (31–37).

More recently, transcriptome sequencing (RNA-seq) was applied to map latency-associated viral transcripts (38). This study revealed a wider viral gene expression profile that included two long noncoding RNAs (lncRNAs), RNA4.9 and RNA2.7, as well as the mRNAs encoding replication factors UL84 and UL44 (38). In a recent study, a targeted enrichment platform was applied to study the transcriptome of HCMV latent infection in both experimental and natural samples, revealing an even broader gene expression profile (39).

Such genome-wide analyses are highly informative as they measure the expression of all transcripts in an unbiased manner. However, a major limitation is that they portray a mean expression in cell population, without reflecting intrapopulation heterogeneity. In the case of latent HCMV infection models, this can be highly misleading since it is hard to exclude the possibility that a small, undesired population of cells is undergoing lytic replication and thus can easily introduce “lytic noise.” This effect can be especially significant for viral genes that are highly expressed during lytic infection, such as lncRNAs (19). Finally, the low frequency of natural latent cells is a major hurdle for global quantitative analysis of naturally latently infected cells.

To overcome the problem of scarcity of natural latent cells, we took advantage of the massive human RNA-seq atlas generated by the Genotype-Tissue Expression (GTEx) Consortium (40). Through analysis of 435 billion RNA reads, we did not find any evidence for a restricted latency-associated viral gene program. Instead, in several tissues we captured low-level expression of viral transcripts that resembles gene expression at late stages of lytic infection. Next, to directly explore viral gene expression in a controlled latently infected cell population, we turned to the established myeloid lineage experimental systems. By using single-cell RNA-seq (scRNA-seq), we unbiasedly characterize the HCMV latency program of both experimentally latently infected CD14^+^ monocytes and CD34^+^ HPCs, overcoming the impediment of cell population variability. Surprisingly, in contrast to the existing view in the field, we find no strong evidence for a specific latency-associated viral gene expression signature of specific viral genes. Instead, we reveal that in HCMV latency models, while there is little detectable IE expression, there is low-level expression of viral genes that largely resembles the late-stage lytic viral gene expression profile. Our analyses thus redefine the HCMV latent gene expression program and suggest mainly quantitative rather than qualitative changes that help determine latency. Our work illustrates how new genomic technologies can be leveraged to reevaluate complex host-pathogen interactions.

## RESULTS

### No evidence for a restricted latency-associated viral gene expression program in natural HCMV infection.

The proportion of infected mononuclear cells in seropositive individuals was estimated at 1:10,000 to 25,000 with a copy number of 2 to 13 genomes per infected cell (41). Given that transcription of viral genes is expected to be low in these cells, an immense amount of sequencing data is required to capture viral transcripts. We thus took advantage of the Genotype-Tissue Expression (GTEx) database, a comprehensive atlas containing massive RNA-seq data across human tissues that were obtained postmortem from otherwise healthy individuals (40). We analyzed HCMV reads in 9,416 RNA-seq samples from 549 individuals covering 31 tissues and containing more than 433 billion reads (see Fig. S1A and B in the supplemental material). In 40 samples, we obtained only reads that aligned with a 229-bp region in the IE promoter (Fig. S1C). Since the sequence in these reads matches the sequence of the HCMV promoter commonly used in vectors rather than the sequence observed in the majority of clinical samples (Fig. S1D), we concluded that these reads may originate from a contamination and excluded them from further analysis.

10.1128/mBio.00013-18.1 FIG S1  Detection of HCMV reads in natural samples. (A) Distribution of the number of total aligned reads per sample in samples from the GTEx data set. (B) Distribution of the number of HCMV aligned reads per sample in positive samples from the GTEx data set. (C) RNA-seq reads from GTEx samples aligned with the MIEP region of the HCMV genome colored by sample. (D) Alignment of RNA-seq reads from GTEx samples and sequences of 101 clinical isolates with the MIEP region (positions 175493 and 175494 in the viral genome). Base variation from the reference (Merlin strain, which is identical in these sites to the CMV promoter that is used in plasmids) is indicated by a color corresponding to the substituting base. The color legend is on the right. (E) Percentage of samples containing HCMV reads in different tissues. Viral reads from samples containing fewer than 2 viral reads were filtered out. (F) Genome browser view showing aligned reads from samples assigned to group I or group II in genome regions coding for abundant genes in these groups. Download FIG S1, EPS file, 2.1 MB. Copyright © 2018 Shnayder et al. 2018 Shnayder et al. This content is distributed under the terms of the Creative Commons Attribution 4.0 International license.

**FIG 1:**
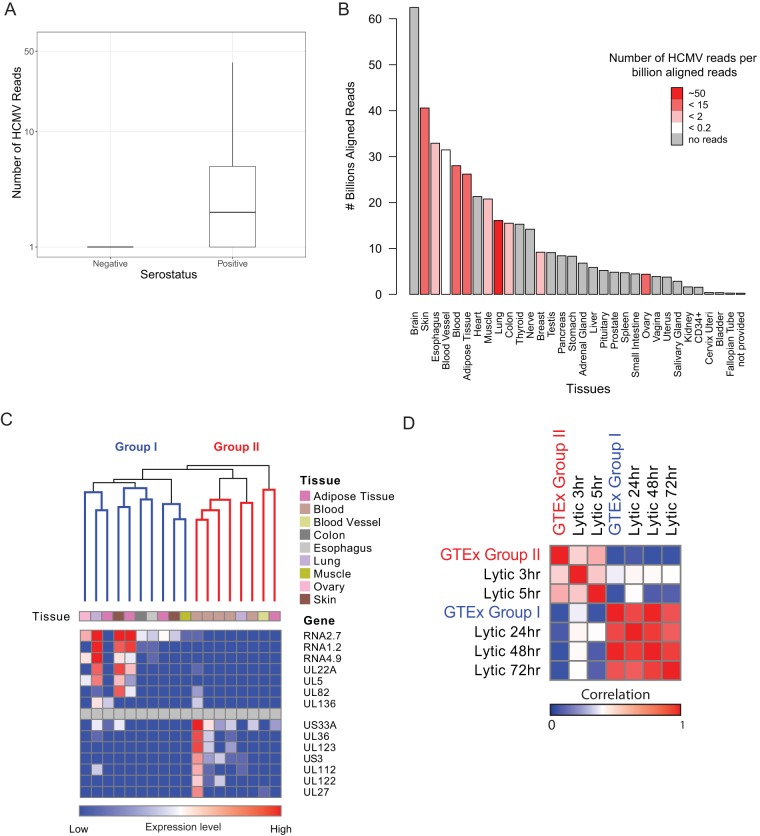
Viral gene expression during natural persistent infection. (A) Box plot showing number of HCMV reads per sample in HCMV-seronegative and HCMV-seropositive samples. (B) Bar plot showing distribution of total sequenced reads in different tissues; color coding reflects the number of viral reads normalized to total number of sequenced reads in each tissue (number of HCMV reads/10^9^ total aligned reads). Viral reads from samples containing fewer than 2 viral reads were filtered out. Data for all samples were obtained from GTEx (40, 74), except for CD34^+^ data, which were collected from 25 different NCBI GEO data sets (Table S1D). (C) Hierarchical clustering of natural samples with more than 4 HCMV reads, according to viral gene expression. The samples are portioned into 2 groups: group I and group II. The upper panel color coding indicates the tissue origin of each sample. The heat map in the lower panel shows the expression level of representative differentially expressed genes in each sample. (D) Heat map showing correlations between viral gene expression program from natural samples from both groups (I and II) and experimental lytically infected fibroblasts at different time points postinfection.

Reassuringly, the number of samples that contained HCMV reads and the number of HCMV reads were significantly higher in samples originating from seropositive individuals (Fig. 1A) (*P* = 0.0467 and *P* < 10^−55^, respectively; hypergeometric test). HCMV reads were found in 6 out of 2,210 seronegative samples; however, all of them contained only one viral read per sample. Therefore, this was used as a threshold, and viral reads from samples containing fewer than two viral reads were filtered out in further analysis (data from all samples are summarized in Table S1A).

10.1128/mBio.00013-18.8 TABLE S1  Analysis of natural infection. (A) Summary of HCMV reads in GTEx samples. Columns indicate sample identifier (ID), subject ID, HCMV serostatus, number of reads (in millions), number of aligned reads (in millions), number of HCMV reads, and number of HCMV reads excluding the MIEP region transcript. Columns I to the end indicate the number of reads for each indicated gene. (B) Attributes of GTEx seropositive samples. The detailed description of what each column represents can be found at ftp://ftp.ncbi.nlm.nih.gov/dbgap/studies/phs000424/phs000424.v7.p2/pheno_variable_summaries/phs000424.v7.pht002742.v7.p2.GTEx_Subject_Phenotypes.var_report.xml. (C) Attributes of GTEx seropositive subjects. The detailed description of what each column represents can be found at ftp://ftp.ncbi.nlm.nih.gov/dbgap/studies/phs000424/phs000424.v5.p1/pheno_variable_summaries/phs000424.v5.pht002743.v5.p1.GTEx_Sample_Attributes.var_report.xml. (D) Analysis of publicly available CD34^+^ RNA-seq data sets. Columns indicate data set ID, sample file ID, cell type, number of reads in indicated sample, and number of aligned reads. Download TABLE S1, XLSX file, 0.1 MB. Copyright © 2018 Shnayder et al. 2018 Shnayder et al. This content is distributed under the terms of the Creative Commons Attribution 4.0 International license.

HCMV genomes have been detected in HPCs and in additional cells throughout the myeloid lineage (42, 43). Consequently, the blood and the hematopoietic system are a major focus in research on HCMV persistence. Analysis of the GTEx database provides an exceptional opportunity to unbiasedly assess HCMV prevalence in various tissues. Interestingly, analysis of the abundance of HCMV reads in different tissues revealed that ovaries, blood, adipose tissue, and lung had the highest percentage of samples containing viral reads (Fig. S1E) as well the highest normalized number of viral reads (Fig. 1B). Since the GTEx database did not contain RNA-seq data from bone marrow, where CD34^+^ HPCs reside, we performed RNA-seq on two CD34^+^ HPC samples from HCMV-positive individuals and surveyed an additional 25 RNA-seq samples of CD34^+^ HPCs from healthy individuals (Table S1D). Although we analyzed over 1.5 billion aligned RNA-seq reads, we did not detect any viral reads in these samples (Fig. 1B).

Next, we analyzed the viral gene expression as reflected by the HCMV reads that we identified in natural samples, including in this analysis only samples that contained more than 4 HCMV reads. Hierarchical clustering revealed that the samples could be subdivided into two groups based on the pattern of viral gene expression (Fig. 1C).

The first group (group I) was composed of samples that were dominated by transcripts that are the most highly expressed during the late stage of lytic infection, e.g., RNA2.7, RNA4.9, RNA1.2, and UL22A (Fig. 1C and S1F). Indeed, when we compared the viral gene expression of these samples to RNA-seq data that we collected during lytic infection of fibroblasts, we obtained a high correlation with late stages of infection (*R* = 0.97) (Fig. 1D and S2A). This correlation suggests that these viral reads that were identified in natural settings resemble the late-stage lytic gene expression program.

10.1128/mBio.00013-18.2 FIG S2  Clustering according to HCMV reads in natural samples. (A) Scatter plot showing read number of viral genes in group I samples from the GTEx database versus lytic fibroblasts 72 h postinfection. (B) Scatter plot showing read number of viral genes in group II samples from the GTEx database versus lytic fibroblasts 5 h postinfection. (C and D) Violin plots showing the time of sample harvesting (measured in minutes after death) versus sample assignment to gene expression group (I or II) (C) and presence or absence of HCMV-specific reads in the sample (D). Download FIG S2, EPS file, 2.3 MB. Copyright © 2018 Shnayder et al. 2018 Shnayder et al. This content is distributed under the terms of the Creative Commons Attribution 4.0 International license.

The second group (group II) is composed of samples that express bona fide immediate early genes, e.g., UL123, US3, and UL36, as well as US33A, which is the most highly expressed transcript early in infection (18), and importantly has very limited expression of transcripts that are abundant at the late stage of lytic infection (Fig. 1C and (S2B). Therefore, we speculate that these samples may reflect the onset of viral reactivation, a state in which IE genes are transcribed but the full viral gene program is still suppressed. Supporting this notion, viral gene expression of these samples correlated best with lytically infected fibroblasts at 5 h postinfection (hpi) (*R* = 0.55) (Fig. 1D and S2B). This IE expression-positive state may represent cells exiting from latency, consistent with the view that reactivation goes through a stage of IE gene activation. Since the tissues that we analyzed were obtained postmortem, it is possible that postmortem-related physiological events led to HCMV reactivation and IE gene expression. To assess this hypothesis, we inspected the time postmortem at which the tissue was collected (data are provided by GTEx [40]). Samples in group II were not enriched for a long waiting time before tissue collection or any other clinical technical details (Fig. S2C and Tables S1B and C). In addition, there were no differences in the time interval of tissue collection between samples that contained HCMV reads and those that did not (Fig. S2D). These results suggest that the HCMV gene expression pattern that we captured is likely independent of the trauma that occurred after death.

Importantly, although we were able to identify HCMV transcripts, we were not able to identify tissue or blood samples that provide evidence for any highly restricted latency-associated viral gene expression program that differs from lytic viral gene expression. Since viral gene expression is expected to be very low in latent cells, a possible explanation for this is that a nontargeted sequencing approach may not detect these rare transcripts despite great sequencing depth.

### Single-cell transcriptomic analysis of latently infected CD14^+^ monocytes.

Although in natural samples we detected only a low-level viral gene expression pattern that resembles the lytic gene expression program, the cellular heterogeneity in these samples does not allow us to distinguish whether we are analyzing latently infected cells or rare cells in which productive infection is taking place. Consequently, we next moved to characterize the viral transcriptome in experimental models of HCMV latency. Since these models rely on primary hematopoietic cells that may vary in their differentiation state and may also contain heterogeneous populations, we took advantage of the emergence of single-cell RNA-seq (scRNA-seq) technologies (44, 45). This high-resolution profiling of single-cell transcriptomes allowed us to delineate the nature of the HCMV latency program in the best-studied latent reservoir, hematopoietic cells.

**FIG 2:**
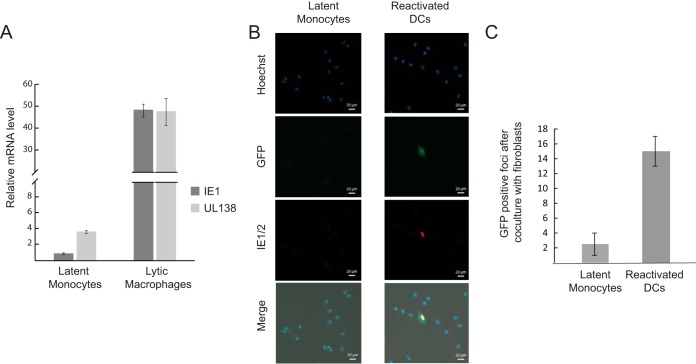
Establishment of HCMV latency in CD14^+^ monocytes. (A) Monocytes and monocyte-derived macrophages were infected with HCMV strain TB40E-GFP at an MOI of 5. RNA was collected at 4 days postinfection (dpi) from the latent monocytes and 5 h postinfection (hpi) from lytic monocyte-derived macrophages and was analyzed by qRT-PCR for the transcript levels of UL138 and IE1. Expression was normalized to the human Anxa5 transcript. Means and error bars (showing standard deviations) represent three measurements. (B) Monocytes were latently infected with TB40E-GFP at an MOI of 5. At 3 dpi, cells were either differentiated into dendritic cells (reactivated DCs) or left undifferentiated (latent monocytes), and 2 days after terminal differentiation, reactivation was visualized by GFP and IE1/2 staining. Representative fields are presented. (C) Monocytes were latently infected with TB40E-GFP at an MOI of 5. At 3 dpi, cells were either differentiated to dendritic cells (reactivated DCs) or left undifferentiated (latent monocytes). Two days after terminal differentiation, cells were cocultured with primary fibroblasts and GFP-positive plaques were counted. The number of positive plaques per 100,000 monocytes or monocyte-derived dendritic cells is presented. Cell number and viability were measured by trypan blue staining prior to plating. Means and error bars (showing standard deviations) represent two experiments.

Freshly isolated CD14^+^ human monocytes were infected with an HCMV TB40E strain containing a simian virus 40 (SV40) promoter-driven green fluorescent protein (GFP) (TB40E-GFP) (46). This strain allows short-term detection of GFP-tagged latently infected cells, as in these cells GFP expression is efficiently detected at 2 days postinfection (dpi) and then GFP signal gradually declines. Despite GFP levels in monocytes being much lower than those in lytic infection, the GFP expression allowed us to confirm that the majority of cells were indeed infected (Fig. S3A). To validate latent infection in our experimental settings, we analyzed by quantitative real-time PCR (qRT-PCR) the gene expression pattern of the well-studied latency-associated gene UL138 and of the immediate early gene IE1 at 4 days postinfection (dpi). Infected monocytes expressed relatively high levels of UL138 while showing only trace levels of IE1 transcript (Fig. 2A), thus manifesting the hallmark of latent infection (29, 31, 32, 37, 47, 48). Differentiation of these infected monocytes into dendritic cells resulted in detectable IE expression as well as production of infectious virions (Fig. 2B and C), thus demonstrating that our CD14^+^ cells are latently infected.

10.1128/mBio.00013-18.3 FIG S3  Validation of infection and scRNA library composition. (A) Flow cytometry analysis showing GFP expression level in population of CD14^+^ monocytes infected with TB40-GFP at 2 dpi. (B and C) Bar plots showing distribution of number of reads per cell (left) and number of genes per cell (right) in scRNA-seq data of infected CD14^+^ monocytes (B) and CD34^+^ HPCs (C). Download FIG S3, EPS file, 1.1 MB. Copyright © 2018 Shnayder et al. 2018 Shnayder et al. This content is distributed under the terms of the Creative Commons Attribution 4.0 International license.

**FIG 3:**
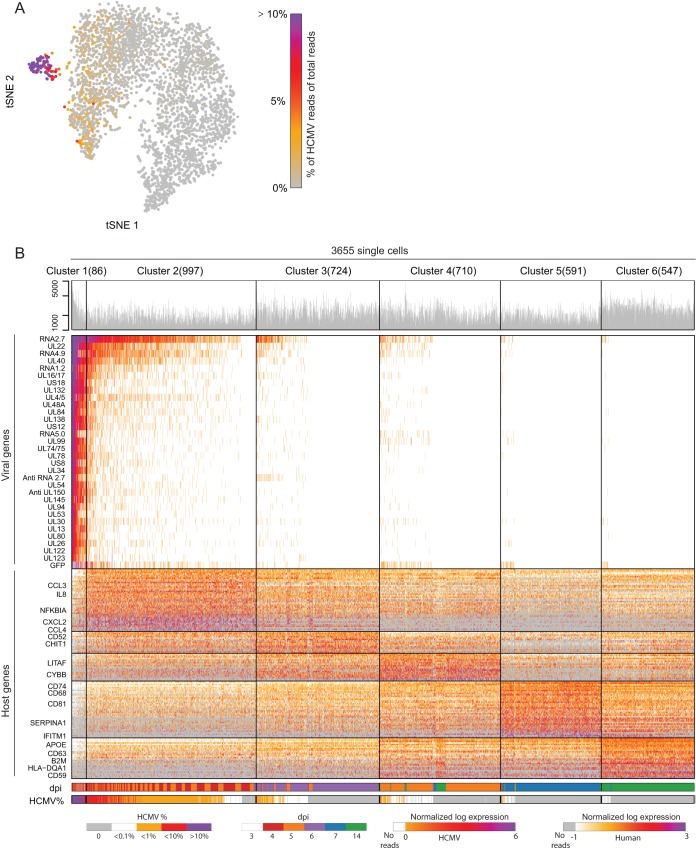
scRNA-seq analysis of latently infected CD14^+^ monocytes. Single-cell RNA sequencing analysis of 3,655 cells from a cell population of latently infected monocytes. CD14^+^ monocytes were infected with HCMV (TB40E-GFP) and analyzed at 3, 4, 5, 6, 7, and 14 dpi. (A) t-SNE plot of all 3,655 single cells based on host and viral gene expression. The color bar shows the percentage of viral reads from total reads per cell. (B) Heat map showing clustering analysis of 3,655 single cells. Rows show expression of the 176 most differential genes (32 out of 171 detected viral transcripts shown in the upper panel, 144 out of 15,812 detected cellular transcripts shown in the lower panel). The bar over the upper panel shows the number of reads obtained for each cell (log scale). Bars under the heat map indicate the percentage of viral reads from total reads and days postinfection for each cell. Cells are partitioned into 6 distinct clusters (1 to 6) based on gene expression profiles and ordered by the relative abundance of viral reads, from high to low. The number of cells in each cluster is shown in parentheses next to the cluster number.

Next, HCMV-infected CD14^+^ cells were single cell sorted without further selection at 3, 4, 5, 6, 7, and 14 dpi, and their transcript levels were measured using massively parallel 3′ scRNA-seq (MARS-seq) (49). Analysis of the entire transcriptome was performed on 3,655 CD14^+^ infected cells, in which we could detect 15,812 genes, out of which 171 were HCMV transcription units (see Materials and Methods and Fig. S3B for distribution of reads and genes over the cell population). Projection of the cells using *t*-distributed stochastic neighbor embedding (t-SNE) analysis revealed that most of the cells constitute a large heterogeneous but continuous population and only a small group forms a distinct population (Fig. 3A). When we calculated the percentage of reads that align with the HCMV genome in each of the cells, it became evident that the viral transcripts constitute >10% of the total reads in the small distinct population (Fig. 3A). Reassuringly, when performing the t-SNE analysis by using only cellular gene expression, we obtained the same structure, confirming that we are looking at two different cell states (Fig. S4A). The small population likely represents a lytic infection state, and the rest of the monocytes, which are the vast majority, exhibit very low to undetectable, diverse viral gene expression levels, indicating that they likely represent latently infected cells. This distribution, showing a clear separation between two groups of cells exhibiting very different levels of viral gene expression, confirms the purity of the single-cell isolation and the dominance of latent cells in the population of CD14^+^ infected cells (Fig. 3A).

10.1128/mBio.00013-18.4 FIG S4  scRNA-seq analysis of latently infected CD14^+^ monocytes. (A) t-SNE plot of all 3,655 single cells based on host gene expression. The color bar shows the percentage of viral reads from total reads per cell. (B) t-SNE plot of 3,655 single latently infected monocytes based on host and viral gene expression (as shown in Fig. 3A) depicting the separation into 6 clusters as shown in Fig. 3B. (C) Scatter plot showing read number of all viral genes in cells from cluster 1 versus lytically infected monocyte-derived macrophages at 4 dpi (left panel) or fibroblasts at 3 dpi (right panel). (D) Scatter plot showing read number of all viral genes in cells from clusters 2 to 6 (labeled on *y* axis) versus cells from cluster 1. Horizontal and vertical error bars indicate 95% nonparametric bootstrap confidence intervals across cells. Download FIG S4, EPS file, 5.2 MB. Copyright © 2018 Shnayder et al. 2018 Shnayder et al. This content is distributed under the terms of the Creative Commons Attribution 4.0 International license.

### HCMV latency-associated gene expression in CD14^+^ monocytes and CD34^+^ HPCs resembles the late lytic gene expression program.

To assess the heterogeneity in HCMV latently infected monocytes, we combined the data from all 3,655 cells and clustered them on the basis of their host and viral gene expression profiles into 6 clusters (Fig. 3B) (clustering method was previously described [50]). Notably, also in this approach, the cells exhibiting high viral expression levels, representing the lytic infection state, were clustered together, and the most differential genes that were highly expressed in this cluster were almost exclusively viral genes (cluster 1, Fig. 3B, top panel). On the other hand, the rest of the cells exhibited very low levels of viral gene expression in various degrees and the highly expressed differential genes in these five clusters were all cellular genes (Fig. 3B, lower panel, and Table S2A).

10.1128/mBio.00013-18.9 TABLE S2  scRNA-seq analysis. (A) Differential genes in latently infected monocytes. Columns indicate gene annotation, the cluster with the highest expression of the indicated gene, expression level of the indicated gene in the cluster where it is most highly expressed (relative to the expression of all other genes in the same cluster), number of reads for the indicated gene in clusters 1 to 6, and total number of reads for the indicated gene across all clusters. (B) Transcripts enriched in latent CD14^+^ monocytes. Columns indicate gene annotation, number of reads in lytic cells (cluster 1), number of viral reads in latent cells (cells in which less than 0.5% of the reads originated from the virus), mean and standard deviation of the number of reads for an indicated gene in the latent cells according to bootstrap analysis (see Materials and Methods), Z-score, and false discovery rate (FDR). (C) Transcripts enriched in latent cells from clusters 2 to 6 compared to lytic cells. Columns indicate gene annotation, number of reads in cluster 1, number of reads in the indicated cluster, mean and standard deviation of the number of reads for each specified gene in the indicated cluster according to bootstrap analysis (see Materials and Methods), Z-score, and false discovery rate (FDR). (D) Viral genes detected in latently infected CD34^+^ HPCs by MARS-seq analysis. The number of reads identified for each of the detected viral genes, in each of the cells. Cell sum- indicates the total number of viral reads per cell. Gene sum- indicates the total number of reads detected for each viral gene. (E) Transcripts enriched in latent CD34^+^ HPCs. Columns indicate gene annotation, number of reads in lytic cells (cluster 1 in CD14^+^ analysis), number of viral reads in infected HPCs, mean and standard deviation of the number of reads for an indicated gene in latent CD34^+^ HPCs according to bootstrap analysis (see Materials and Methods), Z-score, and false discovery rate (FDR). Download TABLE S2, XLSX file, 0.04 MB. Copyright © 2018 Shnayder et al. 2018 Shnayder et al. This content is distributed under the terms of the Creative Commons Attribution 4.0 International license.

**FIG 4:**
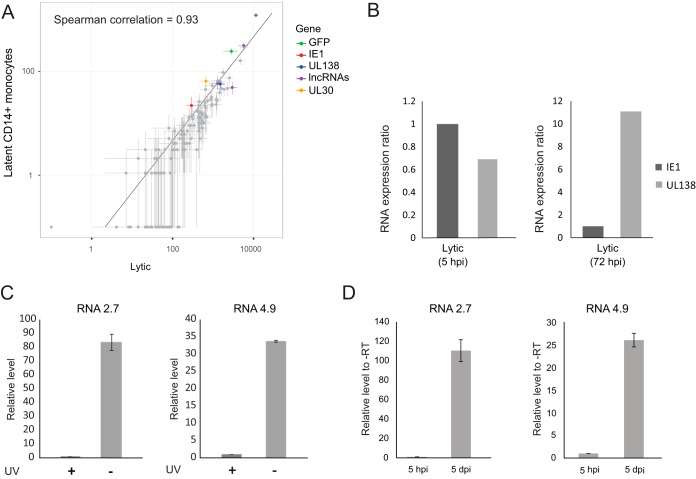
Transcriptional program in latently infected CD14^+^ monocytes. (A) Scatter plot showing read number of viral genes in latent monocytes (defined as cells in which the proportion of viral reads was below 0.5% of total reads) versus lytic cells (cells from cluster 1). Horizontal and vertical error bars indicate 95% nonparametric bootstrap confidence interval across cells. (B) Relative expression of IE1 and UL138 transcripts in RNA-seq data from lytic fibroblasts at 5 and 72 hpi. (C) Relative RNA expression level of viral RNA2.7 (left panel) and RNA4.9 (right panel) in monocytes infected with untreated or UV-inactivated virus, measured by qRT-PCR at 5 dpi. A representative analysis of two independent experiments is shown. (D) RNA expression level of viral RNA2.7 (left panel) and RNA4.9 (right panel), relative to no-RT (-RT) samples, in infected monocytes, measured by qRT-PCR at 5 h and 5 days postinfection. Means and error bars (showing standard deviations) represent three measurements. A representative analysis of two independent experiments is shown.

These clusters were consistent with the t-SNE analysis, with cluster 1 overlapping the distinct population probably representing lytic infection state (Fig. S4B). Indeed, by comparing the viral gene expression pattern of cells from this cluster to that of lytically infected monocyte-derived macrophages or fibroblasts, we could confirm that they exhibit comparable programs (Fig. S4C). Unexpectedly, although the lytic and latent cells represent two very separable cell states (Fig. S4A), latent cells from all clusters show a viral gene expression profile that to a large extent resembles the late lytic expression profile (cluster 1), with the dominant difference being the level of viral gene expression but not the identity of the viral genes (Fig. 4A). The only viral genes whose deviation from this correlation was statistically significant, and which were relatively higher in latent cells, were the exogenous GFP (false discovery rate [FDR], 7 × 10^−19^), which is driven by the strong SV40 promoter; the lncRNA RNA2.7 (FDR, <10^−100^), which is the most abundant transcript; and a transcript encoding UL30 (FDR, 6 × 10^−8^), a poorly characterized coding gene (19) (Table S2B).

We also examined whether the viral gene expression program varies between the different populations of latently infected cells defined by the different clusters, by assessing the correlation between lytic cells (cluster 1) and each of the five other clusters. We found that viral gene expression profiles of all clusters were correlated to some extent with the lytic cells (cluster 1) (Fig. S4D). The correlation coefficient declined with the reduction in number of viral reads, as expected; however, throughout the different clusters only very few viral genes were significantly higher in latent cells composing these clusters (Table S2C).

Interestingly, the continuous decline in viral gene expression appears tightly related to the time during infection and is also reflected in the separation into different clusters (Fig. 3B and S5). This gradual repression suggests progressive silencing of viral gene expression during latent infection as has been previously demonstrated (29, 30).

10.1128/mBio.00013-18.5 FIG S5  scRNA-seq analysis of latently infected CD14^+^ cells clustered by days postinfection. Single-cell RNA sequencing analysis of 3,655 cells from a cell population of latently infected monocytes. CD14^+^ monocytes were infected with HCMV (TB40E-GFP) and analyzed at 3, 4, 5, 6, 7, and 14 dpi. The heat map shows clustering analysis of 3,655 single cells reflecting expression of all viral genes detected. Cells are partitioned into 6 clusters (C1 to -6) according to the day postinfection. The number of cells in each cluster is shown in parentheses next to the cluster number. The bar above the heat map shows the total read number for each cell (log scale). Download FIG S5, EPS file, 1.9 MB. Copyright © 2018 Shnayder et al. 2018 Shnayder et al. This content is distributed under the terms of the Creative Commons Attribution 4.0 International license.

Importantly, by calculating the background noise in the single-cell data (Materials and Methods), we confirmed that the results are not skewed by possible cross contamination in the single-cell data from the few lytic cells that we have in our experiments (Fig. S6).

10.1128/mBio.00013-18.6 FIG S6  Assessment of lytic noise effect on gene expression in CD14^+^ scRNA-seq libraries. Scatter plot showing read number of all viral genes in latent cells (defined as cells in which the proportion of viral reads was below 0.5% of total reads) (A) and in cells from clusters 2 to 6 (labeled on *y* axis) versus cells from cluster 1 (B). Analysis was done after exclusion of cells in which viral read counts were lower than the noise cutoff level (see Materials and Methods). Horizontal and vertical error bars indicate 95% nonparametric bootstrap confidence intervals across cells. Download FIG S6, EPS file, 2 MB. Copyright © 2018 Shnayder et al. 2018 Shnayder et al. This content is distributed under the terms of the Creative Commons Attribution 4.0 International license.

Overall, this analysis indicates that to a large extent the viral gene expression program during experimental latency mirrors the viral gene expression program in the late stage of lytic infection, albeit expressed at much lower levels.

It is noteworthy that these unexpected results do not contradict previous analyses of latent cells, as we observe latent infection to be associated with overall low levels of viral gene expression and with high levels of UL138 relative to IE1. Importantly, this high UL138/IE1 ratio is also evident at late stages but not at early stages of lytic infection (Fig. 4B).

It was previously demonstrated that HCMV virions contain virus-carried mRNAs (51, 52). To exclude the possibility that the transcripts that we capture originate from input mRNAs that are carried in by virions, we infected CD14^+^ monocytes with untreated or UV-inactivated viruses and evaluated the levels of RNA2.7 and RNA4.9 at 5 dpi. The expression of both transcripts was over 30-fold lower in the cells infected with UV-inactivated virus than in cells infected with untreated virus (Fig. 4C). In addition, viral transcripts levels at 5 hpi were much lower than at 5 dpi (Fig. 4D), illustrating that the viral transcripts that we capture during latency result from *de novo* expression and are not the result of input mRNAs.

**FIG 5:**
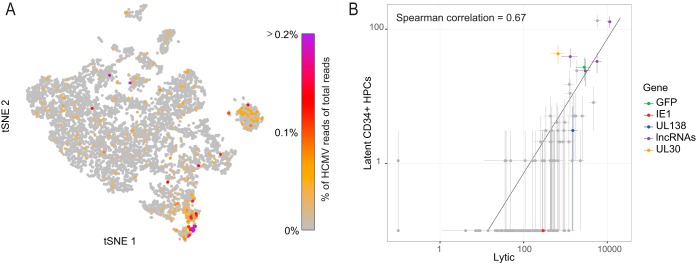
scRNA-seq analysis of latently infected CD34^+^ progenitor cells. Single-cell RNA sequencing analysis of 7,634 cells randomly sampled from a cell population of latently infected HPCs. CD34^+^ HPCs were infected with HCMV (TB40E-GFP) and analyzed at 4 dpi (A) t-SNE projection of all 7,634 single cells based on host and viral gene expression. The color bar shows the level of viral gene expression as a percentage of total reads per cell. (B) Scatter plot showing read number of all viral genes in the latently infected CD34^+^ progenitors versus lytic cells. Horizontal and vertical error bars indicate 95% nonparametric bootstrap confidence intervals across cells.

We next examined viral gene expression in experimentally infected CD34^+^ HPCs, which are another well-characterized site of latent HCMV infection (43, 53). CD34^+^ cells were infected with TB40E-GFP virus in the same manner as CD14^+^ monocytes and used for generation of scRNA libraries at 4 dpi. We initially used MARS-seq (49) to measure the transcriptome of infected HPCs; however, in CD34^+^ cells viral gene expression was significantly lower, and out of 424 cells that we sequenced, viral transcripts could be detected in only 12 cells (Table S2E). We therefore moved to the 10× Genomics Drop-Seq platform that allows simultaneous analysis of thousands of cells. We analyzed the transcriptome of 7,634 experimentally infected HPCs, in 366 of which we identified viral transcripts (see Materials and Methods and Fig. S3C for distribution of reads and genes over the cell population). Projection of cells using t-SNE analysis revealed heterogeneous populations, and cells that expressed viral transcripts were distributed throughout these populations (Fig. 5A). Analysis of the 366 cells that expressed viral transcripts revealed low expression levels, and as in CD14^+^ monocytes, the low viral gene expression that we measured in these cells correlated with the expression pattern of the late stage of lytic infection (comparing CD34^+^ cells to cluster 1, Fig. 5B). Also here, only for a few transcripts, the deviation from this correlation was statistically significant; these included RNA2.7 and UL30 (Table S2E).

Recent transcriptome mapping done on experimentally infected CD34^+^ cells revealed a broader profile of gene expression than was previously appreciated (39). Importantly, comparison of the viral expression profile using this independent data set to the expression profile of late lytic fibroblasts from the same study also revealed significant correlation (*R* = 0.91 and *R* = 0.89) (Fig. S7). Overall, our results and analysis show that during experimental latent infection there is no well-defined latency-associated viral gene expression signature, but rather, these cells are characterized by gradual repression of viral gene expression with low-level expression of a program largely resembling late lytic infection stages.

10.1128/mBio.00013-18.7 FIG S7  Analysis of viral gene expression in latent and lytic samples reported by Cheng et al*.* (39). Scatter plots showing expression of each detected gene in latent (at 6 dpi) versus lytic samples at 48 hpi (left) and 72 hpi (right). *X* and *y* values for each gene represent its percentage out of all viral reads. Values for each gene were calculated as a mean for two donors; error bars indicate standard deviations. Download FIG S7, EPS file, 1.4 MB. Copyright © 2018 Shnayder et al. 2018 Shnayder et al. This content is distributed under the terms of the Creative Commons Attribution 4.0 International license.

## DISCUSSION

Despite the clinical importance of HCMV latency, the mechanisms involved in viral genome maintenance and reactivation are poorly understood. An important step in deciphering these mechanisms is to characterize viral transcripts that are expressed during latent infection in an unambiguous manner. To address this challenge, we examined HCMV infection by comprehensive analysis of RNA-seq data from diverse human tissues and further used scRNA-seq to analyze gene expression of latently infected CD14^+^ monocytes and CD34^+^ HPCs. Surprisingly, our measurements demonstrate that in both natural HCMV infection and experimental latency models there is no evidence of a unique latency-associated gene expression program, but instead, we describe a viral gene expression pattern that is largely similar to the late stage of lytic infection at exceedingly low levels. Although these results are surprising given the prevalent notion that HCMV latency involves a restricted gene expression program, evidence for broader viral gene expression was indicated in several previous genome-wide studies (29, 30, 38, 39).

Examination of HCMV infection by analyzing viral gene expression in diverse human tissues uncovered two patterns of gene expression: the first is composed of samples that contain viral transcripts that are abundant at the late stage of lytic infection, and the second is composed of samples with a restrictive gene expression pattern that includes mainly IE transcripts. The samples that contain late viral transcripts could reflect low-level expression that originates from few latent cells or the existence of scarce lytic cells in these tissues. Since cells expressing viral transcript are very rare, it is currently impossible to distinguish between these two scenarios.

The samples that contained mainly IE transcripts are interesting as they may reflect a snapshot of viral gene expression during reactivation *in vivo*, in natural human samples. Although we did not observe any difference in the time interval from death until these samples were collected, it remains possible that this restricted IE gene expression occurred postmortem or due to the associated trauma (54). Regardless of the conditions that initiated this restrictive IE gene expression, this state may imply that *in vivo* exit from latency goes through a phase in which IE genes are activated. The IE expression pattern that we find was seen mostly in blood samples but not solely. While speculative, the restrictive IE gene expression in these cells may suggest that there is a threshold that needs to be crossed (perhaps the accumulation of enough IE proteins) before the temporally controlled viral gene expression program can start. Indeed, this idea is entirely consistent with differentiation of CD34^+^ cells *ex vivo* to immature dendritic cells (DCs) resulting in cells permissive for IE1 expression but not virus production (11) and with the detection of IE1 expression without infectious virus production in immature DCs isolated from healthy seropositive carriers (55). A similar model was proposed for herpes simplex virus 1 (HSV-1) reactivation from latency, where accumulation and localization of VP16 were suggested to regulate the onset of the full reactivation program (56).

Our analysis of natural samples also suggests that HCMV persistence is widespread throughout the body, as we found viral gene expression in diverse human tissues. Previous studies have shown the presence of viral genomes in tissues outside the blood and hematopoietic system (57–60). Our data provide some evidence for viral gene expression in various tissues. The tissue in which we found the highest levels of viral transcripts was the lung, which is consistent with recent results showing that HCMV DNA could be identified in the lung (60) and in alveolar macrophages (9) and that HCMV reactivation is often manifested clinically as pneumonitis (61, 62). The cellular heterogeneity in tissue samples precludes any conclusion about the cellular sites of HCMV infection in these natural samples.

Our inability to detect a restricted latency-associated gene expression program in this systematic survey of natural samples motivated us to examine the viral gene expression in the best-studied latency experimental systems using single-cell analysis. Notably, our results challenge the view of latency as being a specific virally restricted program and highlight rather a quantitative aspect of viral gene expression that is likely governed by the host cell. Unbiased transcriptome analyses of HPCs and monocytes latently infected with HCMV either experimentally or naturally have been previously performed using both microarrays and next-generation sequencing (29, 35, 38). The list of expressed genes emerging from these different studies included dozens of viral transcripts. The recent study by Cheng et al. (39) revealed an even broader profile of gene expression during hematopoietic cell infection. By using recombinant viruses that establish a latent or a replicative infection in HPCs, this study identified a class of low-expression genes that are differentially expressed in latent versus replicative states of infection and suggested that these genes may have a role in regulating latency. Our analysis of this data set further reveals a significant correlation between viral gene expression in latent HPCs and viral gene expression in late lytic fibroblasts. This correlation provides an important independent validation of our finding that viral gene expression during latency to a large extent resembles the program seen during late-stage lytic infection.

The significant advantage of scRNA-seq, especially in the case of viral infection, is that we can unbiasedly determine the existence of different cell populations and exclude the possibility that the expression profile is skewed by a small group of cells. Importantly, the clustering approach used in this study allows us to validate that the viral gene expression profile is not related to viral expression levels. Although the correlation coefficient is declining with the reduction in the number of viral reads, the decline in viral gene expression level is progressive and suggests continuous repression of viral gene expression during latent infection. Thus, we see expression profiles that correlate with late stages of lytic infection even in the clusters that have almost undetectable levels of viral gene expression.

At the present sampling depth and coverage efficiency, our analysis of CD14^+^ cells can detect subpopulations of 0.3% (11 to 12 cells) or higher. Therefore, although we cannot exclude the possibility that a very small population of cells is in a different state and will harbor a different, more restricted viral gene expression program, if such cells exist they would be rare.

Our analyses reveal differences in cellular gene expression that are associated with differences in the levels of viral gene expression. These differences could stem from variation in the cell maturation state that restricts viral gene expression, or alternatively, they could reflect virally induced changes in the host environment. Future work will help to distinguish between these two options.

The results that we obtained for both CD14^+^ and CD34^+^ progenitors were qualitatively similar; however, the relative levels of viral transcripts in CD34^+^ progenitors were significantly lower, suggesting that these cells are by nature much more repressive. These results are in line with previous studies showing that MIEP is more repressed in CD34^+^ cells (63). Likewise, in natural latency we were unable to detect any viral transcripts by examining more than 1.5 billion RNA-seq reads from CD34^+^ cells. In contrast, by examining 3 billion RNA-seq reads from the blood, we identified 378 viral reads from 18 samples. These results suggest that viral gene expression is more restricted in CD34^+^ progenitors both in natural and in experimental settings and further support the notion that the host cell environment plays a major role in dictating the latency state.

An essential step in understanding HCMV latency is deciphering the importance of viral transcripts and proteins to latency maintenance and to the ability of the virus to reactivate. Based on the view that only a limited number of genes are expressed during HCMV latency, only several candidates for viral functions that may control HCMV latency have been studied. These include UL138 (31, 32), astUL81-82/LUNA (34, 48), UL111A/LAcmvIL-10 (33, 35), and US28 (36, 37). Despite the lack of a clear restricted latency-associated expression program, our results do not undermine the importance of these factors to HCMV latency but rather add many additional candidate genes. Two appealing candidates are RNA2.7 and UL30. RNA2.7 is the most abundant transcript in both lytic and latent cells, but in our measurements, RNA2.7 relative expression in latent cells was constantly higher than expected in comparison to the lytic profile. RNA2.7 was demonstrated to protect infected cells from mitochondrion-induced cell death (64), but its role in latency was never tested. UL30 transcript was suggested to contain *UL30A*, which is conserved among primate cytomegaloviruses and expressed from a nonconventional initiation codon (ACG) (18, 19), but its functional role was never studied. Future work will have to delineate the importance of the different transcripts that we detected to regulating latency.

Overall, our experiments and analyses start to challenge the dogma that all herpesviruses express a highly restricted latency-associated program and suggest that HCMV latency is associated more with quantitative shifts rather than qualitative changes in viral gene expression. Although the relevance of these viral transcripts to latency should be further studied, our findings provide a potential new context for deciphering virus-host interactions underlying HCMV lifelong persistence.

## MATERIALS AND METHODS

### Cells and virus stocks.

Primary CD14^+^ monocytes were isolated from fresh venous blood, obtained from healthy donors, using a Lymphoprep (StemCell Technologies) density gradient followed by magnetically activated cell sorting with CD14^+^ magnetic beads (Miltenyi Biotec).

Cryopreserved bone marrow CD34^+^ cells were obtained from Lonza. Alternatively, fresh CD34^+^ cells were purified from umbilical cord blood of healthy donors. Isolation was done using a Lymphoprep (StemCell Technologies) density gradient followed by magnetically activated cell sorting with CD34^+^ magnetic beads (Miltenyi Biotec). CD34^+^ and CD14^+^ cells were cultured in X-Vivo15 medium (Lonza) supplemented with 2.25 mM l-glutamine at 37°C in 5% CO_2_ (65).

Human foreskin fibroblasts (HFF) (ATCC CRL-1634) and retinal pigmented epithelial cells (RPE-1) (ATCC CRL-4000) were maintained in Dulbecco Modified Eagle Medium (DMEM) with 10% fetal bovine serum (FBS), 2 mM l-glutamine, and 100 units/ml penicillin and streptomycin (Beit-Haemek, Israel).

The bacterial artificial chromosome (BAC) containing the clinical strain TB40E with an SV40-GFP tag (TB40E-GFP) was described previously (66, 67). This strain lacks the US2-US6 region, and therefore, these genes were not included in our analysis. Virus was propagated by electroporation of infectious BAC DNA into HFF cells using the Amaxa P2 4D-Nucleofector kit (Lonza) according to the manufacturer’s instructions. Viral stocks were concentrated by ultracentrifugation at 70,000 × *g* and 4°C for 40 min. Infectious virus yields were assayed on RPE-1 cells.

### Infection and reactivation procedures.

For experimental latent infection models, CD14^+^ monocytes and CD34^+^ HPCs were infected with HCMV strain TB40E-GFP at a multiplicity of infection (MOI) of 5. Cells were incubated with the virus for 3 h, washed, and supplemented with fresh medium. To assess infection efficiency, a sample of the infected cell population was analyzed by fluorescence-activated cell sorting (FACS) for GFP expression at 2 dpi. For single-cell experiments, cells were isolated without further selection; CD14^+^ cells were harvested at 3, 4, 5, 6, 7, and 14 dpi, and CD34^+^ HPCs were harvested at 4 dpi.

Lytic infection was carried out on primary fibroblasts and monocyte-derived macrophages obtained by growing CD14^+^ monocytes in 50 ng/ml phorbol myristate acetate (PMA)-containing medium for 2 days. For reactivation assays, infected monocytes were differentiated into dendritic cells (DCs) at 3 dpi by incubation with granulocyte-macrophage colony-stimulating factor (GM-CSF) and interleukin-4 (Peprotech) at 1,000 U/ml for 5 days, followed by stimulation with 500 ng/ml of lipopolysaccharide (LPS) (Sigma) for 48 h (as previously described in reference 65). Release of infectious virions was assayed by coculturing 100,000 differentiated and nondifferentiated infected monocytes at the end of the differentiation procedure with HFF cells for 10 days and quantification of GFP-positive plaques. Cell number and viability were measured by trypan blue staining prior to plating.

For UV inactivation, the virus was irradiated in a Stratalinker 1800 (Stratagene) with 200 mJ.

### Immunofluorescence.

Cells were fixed in 4% paraformaldehyde for 10 min, permeabilized with 0.1% Triton X-100 in phosphate-buffered saline (PBS) for 10 min, and blocked in 10% normal goat serum in PBS. Detection of IE1 was performed by immunostaining with anti-IE1 antibodies (1:100; Abcam catalog no. ab53495), followed by goat anti-mouse antibody (1:200; Alexa Fluor 647; Invitrogen catalog no. A21235) and Hoechst nuclear stain. Cells were visualized in a Zeiss Axio Observer fluorescence microscope.

### qRT-PCR.

Total RNA was extracted using TRI reagent (Sigma) according to the manufacturer’s protocol. cDNA was prepared using the qScript cDNA synthesis kit (Quanta Biosciences) according to the manufacturer’s protocol. Real-time PCR was performed using the SYBR green PCR master mix (ABI) on a real-time PCR system, QuantStudio 12 K Flex (ABI), with the following primers (forward, reverse): IE1 (GGTGCTGTGCTGCTATGTCTC, CATGCAGATCTCCTCAATGC), UL138 (GTGTCTTCCCAGTGCAGCTA, GCACGCTGTTTCTCTGGTTA), RNA2.7 (TCCTACCTACCACGAATCGC, GTTGGGAATCGTCGACTTTG), RNA4.9 (GTAAGACGGGCAAATACGGT, AGAGAACGATGGAGGACGAC), and Anxa5 (AGTCTGGTCCTGCTTCACCT, CAAGCCTTTCATAGCCTTCC).

### Single-cell sorting and MARS-seq RNA library construction.

Single-cell sorting and library preparation were conducted according to the massively parallel single-cell RNA-seq (MARS-seq) protocol, as previously described (49). In brief, cells from latently infected populations of CD14^+^ monocytes and CD34^+^ HPCs were FACS sorted into wells of 384-well capture plates containing 2 µl of lysis buffer and reverse transcription (RT)-indexed poly(T) primers, thus generating libraries representing the 3′ end of mRNA transcripts. Four empty wells were kept in each 384-well plate as a no-cell control during data analysis. Immediately after sorting, each plate was spun down to ensure cell immersion into the lysis solution, snap-frozen on dry ice, and stored at −80°C until processed. Barcoded single-cell capture plates were prepared with a Bravo automated liquid handling platform (Agilent). For generation of the RNA library, mRNA from cells sorted into capture plates was converted into cDNA and pooled using an automated pipeline. The pooled sample was then linearly amplified by T7 *in vitro* transcription, and the resulting RNA was fragmented and converted into a sequencing-ready library by tagging the samples with pool barcodes and Illumina sequences during ligation, RT, and PCR. Each pool of cells was tested for library quality, and concentration was assessed as described earlier (49).

### RNA sequencing of lytic cells.

For generation of a reference lytic RNA library used in the single-cell experiments, monocyte-derived macrophages or primary fibroblasts were infected with TB40E-GFP virus at an MOI of 5 and used for library preparation at 4 dpi. The libraries were generated from a samples of ~10,000 cells according to the MARS-seq protocol (49).

The lytic fibroblast-derived RNA-seq libraries used as a reference in analysis of the natural samples were previously described (18).

### Single-cell library construction using 10× platform.

Cell suspensions at a density of 700 cells/μl in PBS plus 0.04% bovine serum albumin (BSA) were prepared for single-cell sequencing using the Chromium Single Cell 3′ Reagent version 2 kit and Chromium Controller (10× Genomics, CA, USA) as previously described (68). Briefly, 9,000 cells per reaction were loaded for gel bead-in-emulsion (GEM) generation and barcoding. GEM-RT, post-GEM-RT cleanup, and cDNA amplification were performed to isolate and amplify cDNA for library construction. Libraries were constructed using the Chromium Single Cell 3′ Reagent kit (10× Genomics, CA, USA) according to the manufacturer’s protocol. Library quality and concentration were assessed according to the manufacturer’s instructions.

### Sequencing.

RNA-seq libraries (pooled at equimolar concentration) were sequenced using NextSeq 500 (Illumina), at a median sequencing depth of ~45,000 reads per cell for MARS-seq and ~32,000 reads per cell for the 10× procedure. Read parameters were Read1 (72 cycles) and Read2 (15 cycles) for MARS-seq and Read1 (26 cycles), Index1 (8 cycles), and Read2 (58 cycles) for the 10× procedure.

### MARS-seq CD14^+^ analysis.

The analysis of the MARS-seq data was done with the tools described in references 49 and 50. The reference was created from the hg19 and TB40E (NCBI EF999921.1) strain of HCMV. The transcription units of the virus were based on NCBI annotations, with some changes based on the alignment results. This includes merging several transcripts (taking into account that the library maps only the 3′ ends of transcripts) and adding some antisense transcripts. Read assignment to wells was based on the batch barcode (4 bp) and the well barcode (7 bp) and removal of reads with low-quality barcodes. The read itself (37 bp) was aligned with the reference using Bowtie 2 (69), and the counting of the reads per gene is done based on unique molecular identifiers (UMIs) (8 bp). For each batch, the leakage noise level was estimated by comparing the number of UMIs in the 2 empty wells to the total number of UMIs in the batch. Batches with a high noise level (>8%) were discarded. Wells with <1,000 reads were discarded. The number of wells that were used for further analysis is 3,655. Genes with a low total number of reads (<10) or with low variability (variance/mean of <1.1) and also ribosomal protein and histones were excluded. By using a multiplicative probabilistic model and an expectation-maximization-like optimization procedure, the 3,655 cells were clustered into 6 clusters. The model includes a regularization parameter (=0.5) simulating additional uniform reads to all genes. The clusters are ordered according to the viral content from high to low.

When analyzing correlation in gene expression, the error bars represent 95% confidence intervals that were calculated by 10,000 bootstrap iteration of the cells in each one of the clusters. The t-SNE plot of the MARS-seq CD14^+^ cells was calculated with the R package (70), after down-sampling each cell to 1,000 UMIs.

To exclude background noise, in each one of the batches, all cells with a number of viral reads below 3 times the estimated noise at this batch were excluded.

To estimate the *P* value of getting number of reads *n*, in cluster B, under the null hypothesis of the same expression program as in cluster A, a semiparametric bootstrap method was used. First, the probability of sampling UMIs for each viral gene was calculated according to the gene expression in cluster A. Then, each bootstrap simulation consisted of a parametric step and an aparametric step. The parametric step is, for each cell in cluster B, to sample the number of UMIs according to the actual number of reads in this cell, with distribution over the genes according to the probabilities calculated from cluster A. Then, the aparametric step is a usual bootstrap sampling of the cells in cluster B and calculation of the total number of reads in this cluster B. After doing this simulation 1,000 times, for each viral gene, the mean and the standard deviation of the number of reads in cluster B under the null hypothesis were calculated. Based on this value, the Z-score of the actual value *n* was calculated, and a *P* value was calculated assuming normal distribution of the number of reads under the null hypothesis. Last, these *P* values were adjusted for multiple testing, and only the genes with a false discovery rate (FDR) of <0.01 are reported in Table S2B and C in the supplemental material.

### GTEx and GEO analysis.

All RNA-Seq, paired-end GTEx samples available in July 2016 were used for the analysis. The reference genome that was used was based on hg19 and the Merlin strain of HCMV (NCBI NC_006273.2). Bowtie 2 (69) was used for alignment with the default parameters, besides the additional flag --local. Pairs with a mapping quality of less than 30 were excluded. Pairs with only one read aligned with the Merlin sequence were excluded. For each sample, possible PCR duplications were removed. The counting of the alignments with the genes was done with HTSeq-count (71). Annotation of gff files is based on NCBI data, with some adjustment taking into account correction for the nonstranded library. The clustering for Fig. 1C and D was generated with GENE-E (72). The analysis of the CD34^+^ GEO samples was carried out in the same way. The list of data sets that were used is presented in Table S1D.

### 10× CD34^+^ data analysis.

We used Cell Ranger (73) software with the default settings to process the FASTQ files. The reference was created with the mkref Cell Ranger command, based on the Cell Ranger human hg19 reference and TB40E (NCBI EF999921.1) as was used in the analysis of the MARS-seq data. The demultiplexing of the Illumina files and the analysis were done with the Cell Ranger commands mkfastq and count, respectively. The raw read data were extracted with the Cell Ranger R kit (73). The t-SNE plot is based on the coordinates calculated by the count command.

### Analysis of data from the work of Cheng et al.

The files containing the number of viral reads per samples were downloaded from GSE99823. Full details are given in the work of Cheng et al. (39). Briefly, lung fibroblasts (MRC-5) and CD34^+^ cells from a few donors were infected with the HCMV TB40E strain, and extracted RNA was sequenced (paired end). The computational pipeline includes trimming and quality control (QC) with Trim Galore, alignment with Tophat2, and read counting with HTSeq. In the correlation figure presented, only wild-type samples without any selection were used. For each sample, the number of reads was normalized to the percentage of viral expression, and then for the two CD34^+^ samples, the mean and standard deviation of the percentage were calculated and are displayed in Fig. S7 versus the percent viral expression of the HFF sample.

### Ethics statement.

All fresh peripheral blood samples were obtained after approval of protocols by the Weizmann Institutional Review Board (IRB application 92-1), and umbilical cord blood of anonymous healthy donors was obtained in accordance with local Helsinki committee approval (RMB-0452-15). Informed written consent was obtained from all volunteers, and all experiments were carried out in accordance with the approved guidelines.

### Data availability.

All next-generation sequencing data files were deposited in Gene Expression Omnibus under accession number GSE101341.
